# Patient and public involvement and engagement in co-designing guidance for mental health professionals supporting neurodivergent children and young people in child and adolescent mental health services

**DOI:** 10.1186/s40900-026-00968-4

**Published:** 2026-08-22

**Authors:** Naomi Williams, Helene Torr, Julie Taylor, Helena Tuomainen

**Affiliations:** 1 University of Warwick, Coventry, UK; 2 Notts Parent Carer Voices, Nottingham, UK; 3 University of Birmingham, Birmingham, UK; 4 University of Worcester, Worcester, UK

**Keywords:** Patient and public involvement and engagement, Co-production, Neurodivergent youth, Autism, Child and adolescent mental health services, Mental health service accessibility, Lived experience, Neurodiversity-affirming practice

## Abstract

**Background:**

Neurodivergent children and young people, including those with autism, attention-deficit/hyperactivity disorder and intellectual disability, experience higher rates of mental health difficulties than their neurotypical peers. Despite this increased need, many families encounter barriers when accessing child and adolescent mental health services (CAMHS). These barriers may occur across the service pathway, including referral, triage, assessment, treatment, and discharge. Patient and Public Involvement and Engagement, and professional contributions are essential to ensuring that CAMHS and research reflect the needs and priorities of those who use them. This paper describes a programme of PPIE and professional contribution activities undertaken to co-develop practical guidance for mental health professionals supporting neurodivergent children and young people.

**Methods:**

We – a partnership between a PhD researcher, a parent with lived experience, a young person with lived experience and two PhD supervisors undertook a programme of PPIE activities, using a co-designed ‘Co-production, Accessibility, Participation and Engagement’ approach, over 12 months (December 2024 to November 2025). NW is a doctoral student, and HTr is a PPIE parent and lived experience partner. Together, we facilitated co-production activities with PPIE members, discussions at parent carer conferences, and with professional contributors to support inclusive participation and prioritise ideas. Parents, carers, neurodivergent children and young people, and professionals from health, education, and social care participated in workshops, co-production activities, and discussions exploring experiences of accessing CAMHS and identifying opportunities for service improvement. Insights shared during these activities informed the co-development of practical guidance for mental health professionals.

**Results:**

A total of 60 PPIE and professional contributors took part in co-production activities. Contributors highlighted barriers including communication challenges, misunderstandings of neurodevelopmental differences vs. mental health conditions, fragmented services and limited clinician training in neurodiversity-affirming approaches. These insights informed the development of an open-access guidance resource outlining practical recommendations to support more inclusive communication, service approaches, family participation, and ongoing evaluation of service accessibility.

**Conclusions:**

Collaborative involvement with lived-experience contributors enabled the development of practical guidance to improve accessibility and inclusivity within child and adolescent mental health pathways for neurodivergent children and young people. This work demonstrates how structured PPIE and professional contribution approaches can support the development of resources grounded in lived experience and contribute to ongoing discussions about inclusive child and adolescent mental health service design.

## Abstract (plain language summary)

Neurodivergent children and young people, including those with autism, ADHD and intellectual disability, experience mental health difficulties more often than their peers. Many families report challenges accessing appropriate community mental health services. Long waiting times, limited understanding of neurodivergence, and services designed mainly for neurotypical children and young people can make it harder to receive timely and appropriate support. Patient and Public Involvement and Engagement (PPIE) means working together with people who have lived experience to improve research and services. In this project, parents, carers, neurodivergent children and young people, professionals and researchers worked together to develop guidance for mental health professionals. The aim was to improve how services carry out triage, assessment, treatment and discharge for neurodivergent children and young people. Over 12 months, we held workshops, co-production activities and meetings with lived-experience contributors and professionals from health, education and social care. Participants discussed barriers to accessing services and suggested ways to improve practice. Ideas were discussed and prioritised through structured group discussions. Together, we developed guidance for mental health professionals that outlines practical recommendations for communicating with neurodivergent children, young people, and their families; adapting assessment and therapy approaches; ensuring meaningful participation by families, children, and young people; and evaluating whether services are inclusive. Co-designing guidance with people who use services helps ensure that recommendations are practical, inclusive and responsive to real needs. This work demonstrates how involving families, children, young people, and professionals can contribute to improving accessibility and engagement within mental health services for neurodivergent children and young people.

## Background

Neurodivergent children and young people, including those with autism, attention-deficit/hyperactivity disorder (ADHD) and intellectual or learning disabilities, experience significantly higher rates of mental health difficulties than their neurotypical peers [14]. A systematic review and meta-analysis found high rates of co-occurring mental health diagnoses among autistic individuals, including anxiety, depression or behavioural difficulties [14]. Despite this elevated need, many families report challenges accessing appropriate support within CAMHS.

Barriers can occur at any point along the CAMHS pathway, including referral, triage, assessment, treatment, and discharge [7]. These barriers include long waiting times, fragmented service provision, and lower confidence, knowledge and skills when working with neurodivergent children and young people [3, 7, 10].

Current service models are often designed around neurotypical presentations of mental health conditions and may not adequately account for differences in communication styles, sensory processing or cognitive profiles experienced by neurodivergent children and young people [11]. There is growing recognition that mental health services should adopt neurodiversity-affirming approaches [8]. Neurodiversity-affirming approaches recognise that neurodevelopmental differences are part of natural human variation and emphasise adapting services and interventions to support individuals’ needs [5]. Examples of such adaptations may include providing information in multiple formats, offering flexibility in how appointments are delivered, making sensory adjustments to clinical environments, adapting communication approaches, and involving families and young people in decisions about their care [26].

Patient and Public Involvement and Engagement (PPIE) encompass a range of activities. Engagement typically involves sharing information with communities and raising awareness of research or service development activities, whereas involvement refers to research or service development being carried out with people who contribute their knowledge, experiences and perspectives to shape decisions and outputs [25]. The present project incorporated both engagement and involvement activities to inform the development of guidance for CAMHS professionals.

PPIE is essential in the design of health services and research [9]. Involving people with lived experience can improve the relevance, quality and impact of healthcare initiatives and ensure that priorities reflect the needs of service users [15]. Meaningful involvement can also contribute to more equitable and inclusive service development, particularly for communities that may face barriers to accessing healthcare, such as neurodivergent children and young people, individuals with intellectual disability, and families from underserved or marginalised communities [16].

However, many service frameworks, clinical guidelines and service improvement initiatives are developed using a predominantly top-down approach, whereby priorities and recommendations are determined by policymakers, researchers and professionals with limited involvement from those with lived experience [13]. Such approaches may overlook the practical experiences of families and clinicians navigating services in real-world contexts. Co-production approaches, which involve collaboration between researchers, professionals and people with lived experience, offer an opportunity to develop guidance that is grounded in lived experience and practical knowledge [19].

There is a growing literature describing co-production and PPIE involvement approaches within CAMHS and, more broadly, in mental health research ([13]; [19]). Such approaches have been associated with improved relevance, acceptability and accessibility of services and interventions [25]. However, relatively little attention has been given to involving neurodivergent children and young people, their families, and professionals in the co-development of practical guidance specifically intended to improve accessibility within CAMHS pathways. As a result, there remains a need for examples of how PPIE and professional contributor activities can be used to translate lived experience perspectives into practical resources for service improvement.

This paper describes a programme of patient and public involvement and engagement (PPIE) and professional contributor activities undertaken as part of NW’s doctoral research project, *Autism*,* Intellectual Disabilities and Clinical Practice: Lived Experience Perspectives of Mainstream Child and Adolescent Mental Health Services (AIDE Study)*. This involvement activity aimed to translate lived-experience insights into practical guidance for mental health professionals to improve accessibility and inclusivity within child and adolescent mental health service pathways for neurodivergent children and young people.

## What this paper adds

This paper describes a programme of PPIE and professional contributor activities undertaken as part of a doctoral research project exploring lived-experience perspectives of CAMHS. Through collaborative discussions with parents, carers, neurodivergent children and young people and professionals, this work translated lived-experience insights into practical guidance for mental health professionals.

The paper contributes to the growing literature on co-production and involvement in child and adolescent mental health service design by demonstrating how structured PPIE and professional contributors’ activities can support the development of practical resources intended to improve accessibility and inclusivity within child and adolescent mental health service pathways. In particular, it highlights how collaborative discussions can identify the barriers experienced by neurodivergent children and young people and their families across triage, assessment, treatment, and discharge processes [23].

By documenting the involvement process and its outputs, this paper contributes to methodological discussions about how PPIE and professional contributions can inform child and adolescent mental health service improvement initiatives and support the development of guidance grounded in lived experience [19].

## Methods

Consistent with participatory approaches that emphasise decision-making throughout the research and development process [24], all contributors were involved in identifying priorities, reviewing draft recommendations and refining the final guidance resources. The information provided to contributors explained the purpose of the activities, how feedback would be used to inform guidance development, and the different opportunities for involvement. All contributors received regular updates throughout the project and were invited to review and comment on emerging recommendations and draft versions of the guidance resource.

### Context

We report the patient and public involvement and engagement and professional contributors processes undertaken as part of NWs’ doctoral project, the AIDE Study. The AIDE study is an ongoing doctoral research programme. At the time of writing this paper, the study is ongoing, and further details will be reported separately.

The involvement activities aimed to ensure that lived-experience perspectives informed thinking about how CAMHS could better meet the needs of neurodivergent children and young people.

These activities were part of a structured programme of collaborative involvement. The purpose was to develop practical guidance for professionals and services based on the perspectives of neurodivergent children, young people, their families, and professionals.

### PPIE professional contributors’ approach

The core team comprised NW (doctoral researcher, lived-experience and mental health clinician), HTr (parent lived-experience partner), DP (youth lived-experience partner), and academic supervisors (HT and JT). Team members brought complementary expertise, including clinical research, lived experience of navigating services, youth perspectives, and experience of patient and public involvement and engagement activities. Lived-experience partners were actively involved in identifying priorities, reviewing recommendations and shaping the content and presentation of the final guidance resource [26]. Efforts were made to ensure that different perspectives were valued and incorporated throughout the development process.

We adhered to the principles of co-production and the UK Standards for Public Involvement in Research [20]. To operationalise the project, we developed the Co-production, Accessibility, Participation and Engagement approach (CAPE), at the outset of the project. CAPE was used as a project-specific involvement framework to guide the design, delivery and review of all PPIE and professional contributor activities (see Table 1 for details of CAPE approach).

**Table 1:** CAPE approach used to operationalise PPIE and professional contributor activities

CAPE approach component	How it was applied
Co-production	Shared development of questions and guidance
Accessibility	Advance materials, flexible meeting formats, multiple response options
Participation	Involvement throughout planning, development, and review
Engagement	Ongoing co-production activities, feedback loops and iterative refinement

Our approach was also informed by the 4Pi framework, which emphasises the importance of purpose, presence, process, impact and power-sharing in meaningful involvement activities [25]. These frameworks emphasise inclusive opportunities, collaborative working and meaningful engagement with PPIE and professional contributors. We also adhered to the PPIE reimbursement guidelines set out by Russell et al., [20], reimbursing participants £20 per session for ongoing collaborations or £25 for one-off PPIE engagement. The reporting of this PPIE project was informed by the guidance for reporting involvement of patients and public (GRIPP2) reporting checklist to support transparent reporting of involvement activities and their contribution to guidance development [21].

### PPIE and professional contributors’ overview

We, HTr and NW, spoke to PPIE and professional contributors through established parent-carer networks across three regions of England. Professional contributors were practitioners with experience of working with children and young people in health, education or social care settings. Their role was to contribute professional perspectives on service delivery, review emerging recommendations, and support the development of practical guidance that could be implemented within CAMHS.

### Co-production approach

We facilitated co-production activities using the principles of the nominal group technique, a structured group discussion approach designed to support equal participation and collaborative prioritisation of ideas [27].

The adapted nominal group technique process for online co-production involved:

1. Introducing the question
2. Phase 1: Silent idea generation through individual completion on Microsoft Forms
3. Phase 2: Group discussion of ideas
4. Phase 3: Individual ranking of priorities
5. Phase 4: Further discussion and refinement
6. Phase 5: Final ranking of priorities
7. Phase 6: Discussion and results, and next steps based on the highest-ranked priorities identified by contributors

This adapted process was used across parent/carer and professional activities to identify and prioritise recommendations for inclusion within the guidance.

Within this project, co-production referred to activities in which contributors shared experiences, perspectives and recommendations to inform guidance development. Co-development referred to the collaborative process through which all contributors, and the core team, worked together to review priorities, refine recommendations, shape content and influence the final design and presentation of the guidance resource.

#### Support processes implemented

We ensured that all PPIE members and professional contributors had access to the planned topics for individual and group questions at least one week in advance.

Individual and group meetings were conducted online (MS Teams) and planned at times when PPIE members and professional contributors were available, such as lunchtimes and evenings.

Young people were offered multiple contribution routes, including Microsoft Forms, email and online meetings. The youth’s lived experience partner preferred online meetings and did not request reasonable adjustments. Meeting materials and questions were provided in advance, and opportunities were available to provide feedback verbally, creatively (through drawing) or in writing. Individual meetings with PPIE youth were conducted via online meetings (MS Teams), Microsoft Forms, and email responses, with parents sharing information on behalf of children verbatim, where necessary. Parents were asked to share responses using the child’s own words wherever possible. Some children also attended sections of online meetings, alongside their parents, to hear directly how their feedback would be used. Some children submitted artwork that they had produced as part of their input to the guidance resource; these are displayed within the resource [26].

NW and HTr agreed on an exploratory, broad heading for the PPIE feedback stand at a parent-carer conference, where people could share their thoughts ad hoc throughout the day. The heading was:

CAMHS - Tell Us

The primary question asked during online PPIE and professional contributor discussions was:

What are the essential components for an inclusive assessment, triage, intervention and discharge guidance framework that effectively supports neurodivergent children aged 5–16 and their families in child and adolescent mental health services?

The process involved:

1. Identifying key challenges in accessing services.
2. Generating ideas for improving practice.
3. Sharing and discussing suggestions within the group.
4. Collectively identifying priorities for service improvement.

PPIE youth who contributed through the survey were additionally asked:

What advice would you like to give to staff in mental health services such as CAMHS who work with neurodivergent children and young people?

This approach ensured that PPIE and professional contributors had equal opportunities to share perspectives.

### Organising insights from discussions

As a core team (NW, doctoral researcher; HTr, PPIE lived experience partner; DP, PPIE youth lived experience partner; JT and HT, Supervisors), we then discussed the ideas and perspectives shared during co-production activities.

As these activities were undertaken as PPIE and professional contributor through co-production activities rather than qualitative research, formal qualitative analysis was not conducted. Key points, priorities and suggestions generated during co-production activities were captured through discussion notes, Microsoft Forms responses and written feedback. These were reviewed collaboratively by the core team and contributor groups to identify shared priorities for guidance development. Emerging recommendations were written up, circulated to parents, carers, young people, and professional contributors for feedback, and revised iteratively until consensus was reached on the content and priorities included in the guidance resource [26].

To support this process, the team reflected on insights using the capability, opportunity, motivation behaviour (COM-B) model, which is part of the behaviour change wheel framework [17].

We used the COM-B model as a practical lens to consider different aspects of service improvement, including whether service structures supported inclusive practice, and how attitudes and organisational culture might influence change.

Rather than being used as a formal analytical method, the COM-B model helped organise ideas generated through discussions and supported the translation of PPIE and professional contributors’ lived-experience insights into practical recommendations.

### Summary of co-production activities

Over 12 months, all contributors were actively involved in creating the output rather than solely providing feedback. We conducted a series of co-production activities with parents, carers, neurodivergent young people, and professionals working in mental health, education, and social care. While the AIDE study is an ongoing doctoral research programme, this paper focuses on the development of the guidance resource [26]. The guidance was co-produced through an iterative process involving neurodivergent young people, parents/carers, professionals, and lived-experience partners, who contributed to identifying priorities, generating recommendations, reviewing drafts, refining content, and shaping the final resources, including the design, logo, and presentation. PPIE and professional contributors shared knowledge of CAMHS and discussed potential improvements to service pathways.

Our recruitment plan aimed to include individuals with diverse knowledge of CAMHS. Initial contact with potential PPIE and professional contributors was facilitated through existing network leads and community contacts, who acted as gatekeepers and distributed information about the involvement activities.

## Results

**Fig. 1:**
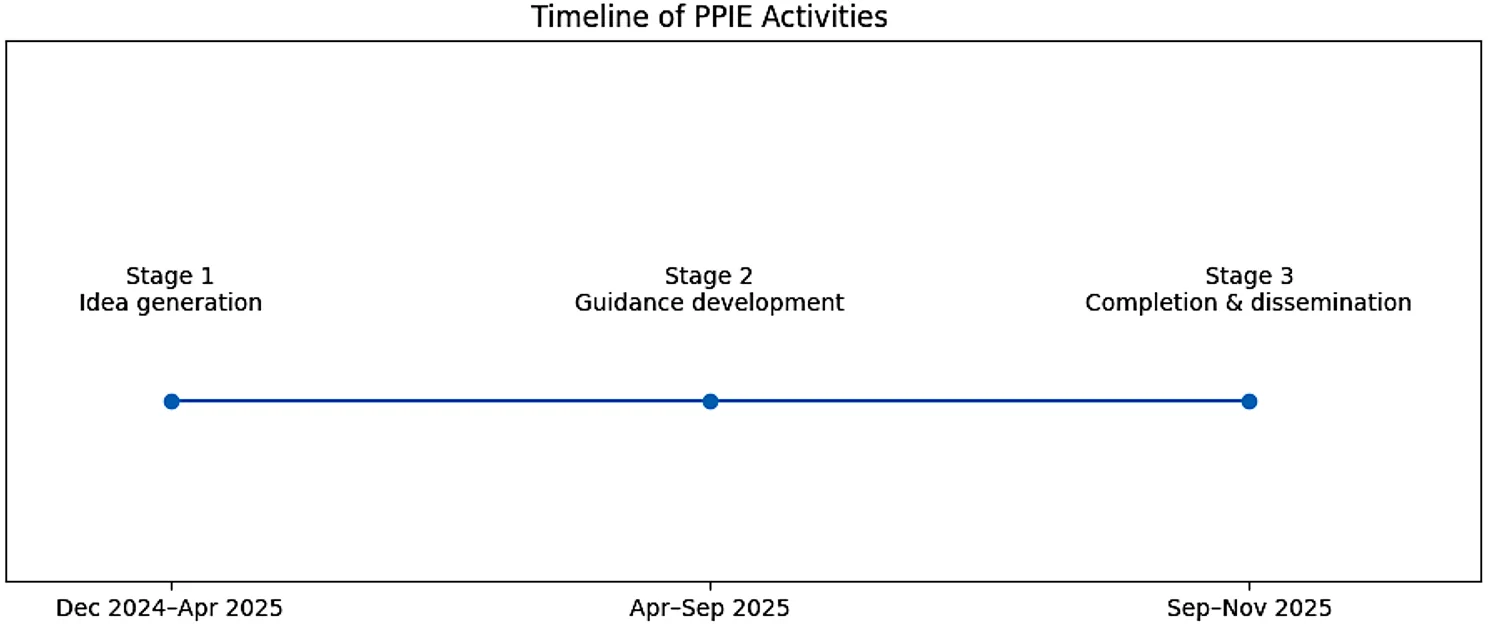
Timeline of PPIE and professional contributors’ activities informing the guidance for mental health professionals and families

The iterative and collaborative nature of the PPIE process is illustrated in Fig. 1.

A total of 60 PPIE and professional contributors were recruited, comprising:

Parents and carers of neurodivergent children (*n* = 6): five mothers and one father. Five were identified as White British and one as Asian British. Participated in online co-production activities via Microsoft Teams. All parent/carers had experience of accessing CAMHS for their child/ren who also had co-occurring neurodevelopmental diagnoses (e.g. autism, ADHD, intellectual disability, and physical health differences).

Neurodivergent children and young people between 12 and 24 years old with experience of mental health services, with parent support (*n* = 28). Twenty-five contributed through a one-off Microsoft Forms questionnaire distributed through youth networks, and no formal demographic information was collected. Three ongoing PPIE youth contributors participated in online co-production activities by sharing feedback with their parents/carers, as this was their preferred method. WhatsApp, online forms, and Microsoft Teams group messaging were also offered. These PPIE youth contributors identified as White British female, Asian British male and Dual Heritage female, and all had experience of accessing CAMHS as a neurodivergent young person.

Professionals with knowledge of CAMHS (*n* = 5), including a social worker (Black British female), a school nurse (White British female), a CBT therapist (White British male), a systemic family therapist (White British female) and a paediatric doctor (Black British female).

One-off contributors of parents, children, young people and professionals at a local parent-carer conference in the East Midlands (*n* = 21). This conference was selected because the PPIE lived experience partner (HTr) was already involved in its organisation, and it provided an opportunity to obtain feedback from parents and carers with lived experiences of navigating CAMHS and related services. All contributors were invited to share anonymous feedback via written comments and post-stick notes at a dedicated information stand. Formal demographic information was not collected for conference attendees.

Several co-production activities took place, these included co-production activities at a parent-carer conference (*n* = 1) and structured online group discussions involving parents/carers (*n* = 8) and monthly email correspondence with updates, neurodivergent young people (via emails from parents and during parent/carer PPIE group meeting) and with professionals (*n* = 10), one meeting for initial information gathering co-production activities and one following for a review of draft guidance for feedback. Online PPIE core group meetings via Microsoft Teams with lived experience partner HTr (*n* = 10), PPIE youth lived experience partner (*n* = 4), and fortnightly email correspondence. NW also discussed via supervision with supervisors (HT and JT) at least once per month. Table 2 summarises the number of PPIE and professional contributor activities.

**Table 2:** Number of PPIE and professional contributor activities

Activity	Quantity
Parent/carer co-production activities	8
Conference co-production activities	1
Professional co-production activities	10
Core group meetings	10
Youth with lived experience meetings	4

A structured timeline of involvement activities is presented in Table 3, which provides an overview of the planning, co-production activities, review and refinement activities undertaken throughout guidance development. The aim was to develop guidance from these activities. Different contributor groups participated in different stages and formats of involvement activities, including ongoing individual and groups discussions, conference-based engagement activities and one-off feedback opportunities. These categories were used to describe how contributors engaged with the guidance development process.

**Table 3:** Timeline of activities: individual and group

Stage	Timeframe	Activities	Purpose
**Stage 1: idea generation**	December 2024	Initial contact between core team members (NW and HTr); introductory meeting established	To initiate collaboration and define scope of PPIE work, CAPE approach developed
	January 2025	Planning meeting with core team	To agree aims, objectives and approach for involving parents/carers, young people and professionals
	January-February 2025	Group co-production activities with PPIE parents/carers	To explore lived experiences
	January 2025	Literature search on existing guidance for mental health support in England	To understand current guidance and identity gaps
	February-March 2025	Core group meetings	To review insights and refine focus of discussions
	March 2025	Individual co-production activities with professionals	To explore existing practices and guidance used in services
	April 2025	Co-development of rationale and summary of key issues	To synthesise insights from discussions and literature
**Stage 2: Guidance development**	April 2025	Ideas, experiences and thoughts shared from PPIE youth advisory group	To incorporate perspectives of neurodivergent young people
	April 2025	Core group meeting to develop questions (adapted nominal group technique)	To structure discussions and support prioritisation
	May 2025	Parent/carer co-production activities at conference	To gather wider perspectives from families
	May-June 2025	Ongoing core group meetings	To review emerging ideas and refine guidance content
	June 2025	Group co-production activities with PPIE parents/carers on draft guidance	To explore relevance and acceptability of proposed recommendations
	June 2025	Individual co-production activities with professionals	To gather feedback on feasibility within services
	August 2025	Review of feedback and co-development of draft guidance	To refine content collaboratively with all contributors
	September 2025	PPIE youth contributors to design (logo and booklet content)	To ensure accessibility and relevance of materials
**Stage 3: Guidance completion and dissemination**	September 2025	Core group review and incorporation of feedback	To finalise guidance content
	October 2025	Final review with PPIE contributors and professionals	To ensure guidance reflects shared priorities
	October 2025	Publication on Open Science Framework and dissemination to NHS Trusts, families and community organisations	To share guidance widely with services and families
	November 2025	Core group reflection and planning for dissemination (e.g. conference submissions, policy engagement)	To support wider impact and future use of guidance

### Development of the guidance

Draft guidance was developed collaboratively by the core team and reviewed during subsequent meetings with PPIE and professional contributors. A PPIE young person designed the cover for the guidance, and two additional PPIE lived experience partners designed the logo. We incorporated feedback into revised versions until PPIE and professional contributors felt that the guidance reflected their experiences and priorities.

The primary output of these involvement activities was an open-access guidance resource for clinicians and families [26].

Table 4 summarises examples of insights shared by contributors, how these were considered using the COM-B framework [17], and how they informed the development of the guidance resource [26].

**Table 4:** PPIE insights and guidance development

COM-B Domain	Barrier/insight identified	Example contributor feedback	Guidance area	How this informed the guidance
Capability	Professionals may misunderstand neurodivergent communication styles	“Talk to me, not at me. Understand I can communicate but not verbally” young person	Communication	Communicate directly with children and young people and offer alternative communication methods
Opportunity	Families not informed about next steps or service processes	“Keep the people involved in a tight loop about what is going on” professional	Engagement and information sharing	Communication and information sharing throughout CAMHS pathway
Motivation	Previous negative experiences reduced trust and willingness to engage with services	“We have been left floating on the water…” parent/carer	Building trust and relationships	Collaborative working, continuity of care and clear discharge planning to support ongoing engagement
Motivation	Feeling listened to and understood supported engagement with services	“The therapist was really listening to me and remembered what I said last time” young person	Neurodiversity-affirming practice	Relationship building, active listening and person-centred care

PPIE and professional contributor feedback were used throughout to inform ongoing refinements to both the process and the final guidance output. Suggestions regarding clarity, accessibility and presentation of the guidance were incorporated into subsequent revisions.

### Reflections from PPIE and professional contributors

PPIE and professional contributors involved in this work emphasised the importance of creating safe and respectful spaces where parents, carers and neurodivergent children and young people felt able to share their experiences of accessing mental health services. PPIE and professional contributors described how discussions provided opportunities to highlight challenges they had encountered while navigating CAMHS pathways, including difficulties during referral, uncertainty about assessment processes, and a limited understanding of neurodivergence within services. Contributors described how these challenges affected their experiences in several ways, such as feeling unheard, misunderstood, or excluded from decision-making.

PPIE and professional contributors noted that developing practical guidance felt meaningful, as their experiences could inform recommendations to improve clinical practice. Parents highlighted that lived experience can provide insights that may not always be visible within policy or service documentation. Neurodivergent children and young people emphasised the importance of clear communication, flexibility within services and clinicians taking time to understand individual needs.

Professional contributors also noted that involvement processes should remain accessible and inclusive. PPIE and professional contributors considered practical, reasonable adjustments such as clear language, flexible meeting formats, and opportunities to contribute in different ways, which are important for enabling participation.

Overall, PPIE and professional contributors noted that collaborative discussions among families, children, young people, and professionals helped create guidance grounded in real experiences with services. These reflections reinforce the value of involving PPIE and professional contributors when developing resources intended to support improvements in mental health services.

### PPIE and professional contributors impact report

Contributors were invited to provide feedback regarding their experiences of involvement, perceived impact of their contributions, and reflections on the developing guidance resource. The evaluation of the PPIE and professional contributor process and guidance resource was undertaken through a PPIE and professional contributors’ impact report completed by a subset of contributors (*n* = 8). Respondents reported valuing the opportunity to contribute to guidance development, for example, one parent reflected on “Knowing we can help neurodivergent families have a more positive experience in the future”. While a young person highlighted their role in “Developing and writing the practical guide for professionals and parents”. Contributors also valued hearing different perspectives and felt their own views were listened to. One contributor also shared, “I’m more interested in research”. Overall contributors confirmed that they could see “a lot of” their input in the guidance resources “Contributing to development of Making Mental Health Services Better for Neurodivergent Children and Families: A practical guide”.

## Discussion

A strength of this work was the use of the CAPE approach, which provided a practical framework for supporting co-production, accessibility, participation, and engagement throughout the development of the resource guidance.

A key principle underpinning this work was the recognition that people with lived experience hold valuable expertise regarding how services operate in practice [22]. By involving parents, carers, and neurodivergent young people in discussions about service pathways, opportunities emerged to shift traditional power dynamics between professionals and service users [6]. Rather than positioning all contributors solely as consultees, the approach aimed to foster collaborative dialogue in which lived experience informed practical recommendations for mental health professionals.

Power sharing in PPIE is increasingly recognised as essential for meaningful involvement, particularly when working with communities whose perspectives are historically underrepresented in health service design [12]. We involved PPIE and professional contributors throughout the planning, design, and review of the guidance approach, which helped ensure that the resulting recommendations reflected priorities identified by families and neurodivergent young people rather than being developed solely from research perspectives.

At the same time, achieving equitable power sharing within involvement activities can be challenging [6]. Contributors may have different levels of familiarity with child and adolescent mental health service pathways, and professionals may hold implicit authority within discussions. We made efforts to facilitate inclusive discussions, provide clear explanations of terminology and ensure that all PPIE and professional contributors had opportunities to share their perspectives [25]. These considerations align with broader recommendations for meaningful involvement in health research and service improvement [1].

### Strengths and challenges of PPIE and professional contributions

The CAPE approach may offer a useful framework for future PPIE activities involving neurodivergent children, young people, families and professionals by providing a practical structure for embedding co-production, accessibility, participation and engagement throughout guidance development.

A key strength of this work was the inclusion of perspectives from multiple stakeholders, including parents, carers, neurodivergent children and young people and professionals with knowledge of health, education and social care. This diversity of perspectives enabled the identification of barriers across the service pathway, from referral and triage through to treatment and discharge.

Collaborative approaches help ensure that guidance reflects practical realities faced by both service users and practitioners. In this case, discussions highlighted issues that might not always be evident in policy documents or clinical guidelines, such as communication barriers, uncertainty during referral processes, and the impact of misunderstanding neurodevelopmental presentations on access to mental health support. Communication barriers described by some contributors included difficulties understanding clinical language, limited opportunities to communicate using preferred methods, and experiences of professionals speaking about rather than directly to neurodivergent children and young people. Contributors highlighted that neurodivergent presentations were sometimes misunderstood as non-engagement, oppositional behaviour, or lack of motivation to participate in treatment. Such misunderstandings may contribute to delays in accessing appropriate support, reduced engagement with services, and poorer experiences of CAMHS. Bringing together lived experience and professional perspectives helped generate recommendations that PPIE and professional contributors found realistic and achievable within existing services. Recommendations focused on improving communication, increasing flexibility within service delivery, recognising and responding to neurodivergent presentations, involving families in care planning, and providing clearer support during discharge and transition processes.

However, collaboration also presents challenges. Coordinating discussions across different stakeholder groups requires time, resources and careful facilitation. PPIE and professional contributors may have varying expectations about the goals and outcomes of involvement activities, and balancing these perspectives can be complex. Contributors held differing expectations regarding the scope of the guidance, the types of service changes that could realistically be implemented, and the extent to which recommendations should focus on individual clinical practice rather than wider service-level changes. Additionally, PPIE and professional contributor activities, such as parent and carer co-production activities groups, professional co-production activities meetings, youth feedback activities, conference-based co-production activities, and review of draft guidance materials, took place as part of ongoing research and clinical responsibilities, which can affect the availability of PPIE and professional contributors and facilitators.

Despite these challenges, PPIE and professional contributors reported that having opportunities to share experiences and contribute to guidance development was valuable. The collaborative process also helped build relationships between PPIE and professional contributors, which may support future involvement and service improvement initiatives.

### Implications for child and adolescent mental health policy and service design

The discussions undertaken during this work highlight several areas where CAMHS may benefit from increased attention to the needs of neurodivergent children and young people. PPIE and professional contributors consistently emphasised the importance of clear communication, flexible service approaches, and meaningful family involvement in decision-making [7, 13].

These priorities align with broader policy discussions on the need for more inclusive, person-centered mental health services [4, 18]. Neurodiversity-affirming approaches are increasingly recognised as important within clinical practice, emphasising the need to adapt services to accommodate diverse communication styles, sensory needs and cognitive profiles. PPIE and professional contributors highlighted that relatively small changes may have a significant impact on accessibility for neurodivergent children and young people [2]. For example, professionals providing clearer information about referral processes, the importance of adapting appointment structures to better meet individual needs [26]. Examples included providing flexibility in appointment length and format, offering opportunities for breaks, sharing information in advance, considering sensory needs, and allowing neurodivergent children and young people and families to choose how they engage with services.

The guidance developed through this work may therefore contribute to ongoing discussions about improving CAMHS pathways for neurodivergent populations. The guidance will not replace existing clinical frameworks, but can offer practical considerations to support services in reflecting on how inclusive their processes are across triage, assessment, treatment, and discharge.

Formal service evaluations may not capture the priorities of neurodivergent young people and their families. However, actively involving neurodivergent young people and their families in discussions about service design can improve service quality and offer lived experience perspectives. This work demonstrates how structured PPIE and professional contributor activities can contribute to service improvement initiatives.

### Alignment with guidance for reporting involvement of patients and the public 2 reporting standards

This paper has been reported in line with the GRIPP2 (Guidance for Reporting Involvement of Patients and the Public) reporting checklist, developed to improve transparency and consistency in reporting patient and public involvement in research [21]. Key elements recommended by GRIPP2 include describing the aims of involvement, the methods used to engage all contributors, and the impact of involvement on outputs or processes.

In this paper, involvement aimed to ensure that lived-experience perspectives informed the development of practical guidance for mental health professionals working with neurodivergent children and young people.

The primary outcome of these involvement activities was the development of an open-access guidance resource designed to support more inclusive practice within child and adolescent mental health service pathways. Reporting these processes contributes to the growing literature describing how PPIE and professional contributors can inform service design. It highlights the value of collaborative approaches when developing practical guidance for clinical settings.

Future work should focus on evaluating the implementation, acceptability and impact of the guidance within CAMHS settings. Further refinement of the resource through ongoing collaboration with neurodivergent children and young people, families and professionals may support wider adoption and continued service improvement.

## Limitations

Several limitations should be considered. Contributors were recruited primarily through existing networks and may therefore not represent the experiences of all neurodivergent children, young people, families, or professionals. Demographic information was not collected consistently across all involvement activities, limiting the extent to which contributor diversity can be described. Younger children were underrepresented, with some feedback communicated through parents and carers. In addition, these activities were conducted as PPIE rather than research and therefore did not involve formal qualitative data collection or analysis procedures. Future work could build on this guidance by expanding involvement activities and conducting a formal evaluation of its implementation and impact.

## Conclusion

This paper describes a programme of patient and public involvement and engagement activities undertaken as part of the AIDE doctoral study, which aimed to ensure that lived-experience perspectives informed the development of practical guidance for mental health professionals working with neurodivergent children and young people. Through collaborative discussions involving parents, carers, neurodivergent children, young people and professionals, contributors shared experiences of navigating CAMHS. They identified barriers that can arise throughout referral, triage, assessment, treatment and discharge processes.

The involvement process highlighted several priorities for improving accessibility within services, including clearer communication, flexible service approaches, meaningful family participation in decision-making, and ongoing reflection on how inclusive services are for neurodivergent individuals. These insights informed the development of guidance for mental health professionals [26], an open-access resource intended to support clinicians and services in reflecting on their practices and considering practical adaptations.

This work demonstrates how structured PPIE and professional contributor activities can contribute to the development of resources grounded in lived experience. Bringing together families, children, young people, and professionals created opportunities for dialogue about service challenges and potential improvements. While the guidance will not replace existing clinical frameworks, it offers practical considerations that may support services seeking to improve accessibility and engagement for neurodivergent children and young people.

Future work may explore how guidance developed through PPIE and professional contributors can inform service improvement initiatives and contribute to wider discussions about inclusive mental health care for neurodivergent populations.

## Data Availability

No datasets were generated or analysed during the current study.

## Abbreviations

ADHD: Attention Deficit Hyperactivity Disorder
CAMHS: Child and adolescent mental health services
PPIE: Patient and public involvement and engagement
GRIPP2: Guidance for Reporting Involvement of Patients and the Public
